# Effects of a 4-week vinyasa yoga intervention on sleep and cardiovascular health in adults with insomnia symptoms: a pilot randomized controlled trial

**DOI:** 10.1093/sleepadvances/zpag107

**Published:** 2026-10-07

**Authors:** Caitlin A Cheruka, Sally A Sherman, Tyler D Quinn, Elizabeth G Giordano, Sophie M Lebegern, Henry Matthew Lehrer, Daniel J Buysse, Kelliann K Davis, Christopher E Kline

**Affiliations:** Institute of Exercise Physiology and Rehabilitation Science, University of Central Florida, Orlando, FL, United States; Health and Human Development Department, University of Pittsburgh, Pittsburgh, PA, United States; Epidemiology and Biostatistics, West Virginia University, Morgantown, WV, United States; Health and Human Development Department, University of Pittsburgh, Pittsburgh, PA, United States; Health and Human Development Department, University of Pittsburgh, Pittsburgh, PA, United States; Health and Human Development Department, University of Pittsburgh, Pittsburgh, PA, United States; Health and Human Development Department, University of Pittsburgh, Pittsburgh, PA, United States; Health and Human Development Department, University of Pittsburgh, Pittsburgh, PA, United States; Health and Human Development Department, University of Pittsburgh, Pittsburgh, PA, United States

**Keywords:** actigraphy, insomnia symptoms, physical activity, yoga, cardiovascular health, randomized controlled trial

## Abstract

**Study Objectives:**

Vinyasa yoga (VY) yields moderate-intensity physical activity that may improve sleep and cardiovascular health (CVH). However, evidence is scarce regarding the effects of VY on CVH. This study examined the effects of a 4-week VY intervention on sleep and CVH in adults with insomnia symptoms.

**Materials and Methods:**

A total of 33 insufficiently active adults (84.8 per cent female; 78.8 per cent White; 34.9 ± 10.6 y; 28.9 kg/m^2^) with insomnia symptoms (Insomnia Severity Index [ISI] ≥10) were randomized to a 4-week VY intervention (*n* = 17) or non-active control (CON; *n* = 16). VY participants practiced 3×/week following pre-recorded 60-minute videos and CON maintained their current lifestyle. Resting daytime blood pressure (BP), heart rate (HR), and HR variability (HRV) were obtained at baseline and post-intervention. Sleep was measured using the ISI, actigraphy, and a sleep diary. One night during sleep assessment, participants wore a chest HR monitor to assess HRV; primary HRV outcome was root mean square of successive differences. Analyses used linear mixed models and Cohen’s *d* effect sizes.

**Results:**

Changes in ISI, actigraphy-, and diary-assessed sleep from baseline to post-intervention did not differ between groups (each *p* > .06). Changes in nocturnal HRV and daytime resting BP and HRV were not significant (each *p* ≥ .30). Resting HR decreased for VY (72.41 ± 2.92 to 69.50 ± 2.60 bpm) and increased for CON (70.47 ± 2.54 to 72.93 ± 2.60 bpm) (*p* = .047). Additionally, among highly adherent participants, VY reduced ISI scores more than CON (*p* = .002).

**Conclusion:**

VY improves insomnia symptoms among those who practice regularly. It does not appear to improve nocturnal cardiovascular physiology, and shows modest benefits on daytime cardiac parasympathetic activity.

**Clinical Trial Registration:**

Registry: ClinicalTrials.gov; Name: Yoga for Insomnia Study; URL: https://clinicaltrials.gov/study/NCT05723211; Registration ID: NCT0572321; Date of Registration: January 2, 2023.

## Teaser

**Statement of Significance** This study presents preliminary findings on the short-term effects of vinyasa yoga (VY) (a moderate-intensity style of yoga), showing a significant reduction in insomnia severity among highly adherent participants. Additionally, VY significantly reduced resting heart rate following the intervention. These findings provide preliminary evidence of the potential of VY as a complementary therapy for improving insomnia symptom severity. Future studies should focus on increasing adherence to encourage regular yoga practice. Furthermore, longer interventions and comparisons of VY with other styles of yoga will contribute to understanding the unique benefits, potential limitations, and overall impact on insomnia disorder and sleep outcomes. This may guide clinicians on which style(s) are most suitable for sleep and cardiovascular health outcomes.

## Introduction

Insomnia is the most prevalent sleep condition in the general population and can present as a symptom, disorder, or both [1]. While prevalence estimates vary widely, most studies suggest that approximately one-third of the US population reports at least one insomnia symptom, and nearly 12.4 percent fulfills the diagnostic criteria for insomnia disorder [1]. The prevalence of insomnia symptoms is important not only because of the distress and impairment it causes, but also because of the well-established association between insomnia and cardiovascular disease (CVD). The risk of developing CVD or cardiovascular mortality is approximately 45 per cent higher in individuals with insomnia compared to those who are good sleepers [2]. Insomnia is also associated with subclinical cardiovascular risk factors such as hypertension, elevated resting heart rate (HR), and reduced heart rate variability (HRV) [3]. Thus, treating insomnia may help to reduce CVD risk.

While cognitive-behavioral therapy for insomnia is the consensus first-line treatment for insomnia, its availability remains limited and its impact on cardiovascular biomarkers is limited [4–6]. These limitations have led to the exploration of alternative and complementary treatments for insomnia, such as physical activity. Physical activity has consistently been shown to improve sleep in healthy adults and adults with sleep disturbances (e.g. insomnia, obstructive sleep apnea) [7]. However, the majority of research on physical activity and insomnia has focused on traditional modes of exercise, such as aerobic (e.g. walking, cycling) or resistance exercise. Therefore, it is important to determine whether other potential alternative and/or complementary modes of physical activity have similar benefits on sleep and insomnia.

One such mode of physical activity that holds promise is yoga, particularly vinyasa yoga (VY). This form of yoga utilizes breath-synchronized movement to establish a continuous flow between certain poses, specifically at the start of the yoga practice [8]. VY has been shown to improve cardiovascular health (CVH) [9, 10], flexibility [11], strength [12], and mood [13]. Moreover, prior research has demonstrated that VY satisfies the requirements for moderate-intensity physical activity [14, 15]. VY may have the potential to yield comparable or potentially superior effects on sleep compared to traditional forms of physical activity. This may be attributed to its status as a mind–body practice since yoga incorporates mind–body awareness with deep relaxation components in conjunction with moderate-intensity physical activity [14, 16]. Through slow and rhythmic breathing, yoga stimulates the vagus nerve and increases parasympathetic nervous system activity [8]. Regular yoga training has been shown to increase high-frequency HRV, a marker of parasympathetic nervous system activity [17]. In addition, VY may improve regulation of the sympathetic nervous system and hypothalamic–pituitary–adrenal axis potentially through adaptations associated by regular physical activity [18, 19]. There is growing evidence that suggests sleep is associated with CVD [20] and physical activity is widely touted as one of the most effective strategies in primary and secondary prevention of CVD [21]. Therefore, the combination of moderate-intensity physical activity and breathing techniques within VY may work to dampen the hyperarousal associated with insomnia disorder by normalizing activity of both branches of the autonomic nervous system and the hypothalamic–pituitary–adrenal axis, potentially leading to improved sleep and CVH.

To our knowledge, no study has examined the impact of VY on sleep in adults who report insomnia symptoms, as previous studies have primarily focused on low-intensity yoga styles. Therefore, the purpose of this randomized controlled trial was to examine the effects of a 4-week VY intervention on sleep and CVH in adults experiencing insomnia symptoms. Our primary hypothesis was that, compared to a non-active control condition, VY would significantly decrease insomnia symptoms. We also hypothesized that VY would significantly increase nocturnal HRV compared to a non-active control condition.

## Materials and methods

### Study design

This study was a single-centered, 2-arm, parallel-group, randomized controlled trial conducted at the Physical Activity and Weight Management Research Center in Pittsburgh, Pennsylvania. The study was approved by the University of Pittsburgh Institutional Review Board (STUDY22110168) and registered as a clinical trial on February 1, 2023, through clinicaltrials.gov (NCT05723211: https://clinicaltrials.gov/study/NCT05723211). Data recruitment and collection occurred between March and June of 2023. All eligible participants provided written informed consent prior to study commencement.

### Participants

Thirty-three adults (18–55 years) who were insufficiently active (i.e. below public health physical activity guidelines assessed by Stanford Leisure-Time Activity Categorical Item) [22] and who had insomnia symptoms were recruited into the present study. Insomnia symptoms were assessed using the Insomnia Severity Index (ISI); participants were eligible with scores ≥10, which has been shown to be the optimal cut-off point for detecting insomnia [23]. Exclusion criteria included regular vinyasa and/or power yoga practice; diagnosed medical condition requiring medical clearance; physical limitations and/or mobility restrictions; untreated major psychiatric disorders; high risk for sleep apnea; current or planned pregnancy within the next 3 months; current treatment for insomnia (e.g. cognitive behavioral therapy for insomnia [CBT-I], pharmacological therapies); overnight shiftwork; and medication that may affect HR during exercise. Participants taking medications for depression, anxiety, or other medical conditions were not excluded from the study. Participant recruitment occurred via word of mouth, flyers, and advertising on the University of Pittsburgh Clinical and Translational Science Institute’s research registry platform (Pitt+Me).

Following determination of initial eligibility, participants visited the research facility to complete a demographic and medical history survey, Physical Activity Readiness Questionnaire for Everyone [24], and STOP-Bang screening for sleep apnea (including assessment of body mass index and neck circumference) [25]. Individuals were excluded from the study if physical activity was deemed to be unsafe without medical approval or if they screened as “high risk” for sleep apnea (i.e. STOP-Bang score ≥5) [25]. Participants who remained eligible after these additional screening procedures were encouraged to complete baseline experimental assessments.

### Experimental procedures

#### Outcome assessments

Baseline assessments of sleep and CVH were performed over an 8-day period prior to the intervention. These procedures were repeated following the 4-week intervention.

##### Sleep

Participants were given an Actiwatch Spectrum Plus (Philips Respironics; Bend, Oregon) accelerometer to assess behavioral sleep/wake patterns. Participants were instructed to wear the Actiwatch on their non-dominant wrist for 8 consecutive days. The Actiwatch collected accelerometry, ambient light, and event marker data in 30-second epochs. Accelerometry wear was supplemented by daily completion of the Expanded Consensus Sleep Diary for Evening [26]. In addition, participants were provided with a Bluetooth-enabled HR monitor (Polar H10; Bethpage, New York) for one night of continuous HR measurement on a typical night of sleep.

##### Cardiovascular health

During one morning between 7:00 and 11:00 hours during the week of sleep assessment, participants reported to the research facility for assessments of daytime CVH. Participants were instructed to fast overnight, refrain from alcohol, and moderate- or vigorous-intensity physical activity during the 24 hours prior, and abstain from nicotine and caffeine that morning. Standardized resting measurements of blood pressure (BP), HR, HRV, and body composition were obtained.

Resting systolic and diastolic BP assessments were obtained using an automated BP system (Dinamap Carescape V100 Monitor; GE Healthcare, Chicago, Illinois) on the right arm in the seated position, with back supported and feet flat on the ground, after a 10-minute rest period. Two BP measurements were taken with at least 1 minute between each measurement. If systolic and/or diastolic BP differed by ≥10 or ≥6 mmHg, respectively, a third measurement was taken [27]. Participants then assumed the supine position on a padded table for a 10-minute rest period, followed by a 6-minute HR recording using a Polar H10 HR monitor that was paired via Bluetooth with an ActiGraph GT9X Link (ActiGraph Corp, Pensacola, Florida); HR was continuously measured and stored on the GT9X Link at a beat-to-beat resolution of 1 millisecond. Following the HR measurement, participants underwent bioelectrical impedance analysis (BIA) to assess body composition using the Quantum II (RJL Systems, Clinton, Michigan). Finally, participants completed the ISI (along with several questionnaires not described here).

#### Randomization

Following baseline assessments and prior to the initial supervised intervention session, participants were randomly assigned to one of two experimental conditions, VY or non-active control condition, stratified by sex in a 1:1 ratio. Randomization sequences were generated using Statistical Analysis System (SAS) v 9.4; Cary, North Carolina) using blocks of four with equal allocation to the two experimental conditions. A research staff member, otherwise unaffiliated with the data collection, prepared the randomization assignments in opaque envelopes.

Participants were notified of their randomization assignment following the completion of baseline assessments at the initial experimental session. The research staff member opened the sealed opaque envelope in front of the participants to notify them of their experimental condition they had been assigned.

### Intervention

Participants reported to the research facility for the initial intervention session. Details of the initial session have been published elsewhere [28]. Briefly, participants completed an initial 60-minute supervised session that began between 15:00 and 19:00 hours and consisted of either VY (in which participants followed a pre-recorded video for standardization) or quiet rest (in which participants watched a nature documentary). All other intervention sessions were completed remotely. There were no adaptations to the study intervention. The intervention duration was the same for both treatment groups.

#### Vinyasa yoga condition

Participants were provided with a yoga mat for home-based practice. The VY sequence utilized for this study was the “Journey into Power” sequence from Baptiste Power Yoga [29], which has been shown to meet the criteria for moderate-intensity physical activity [14, 15]. Details of the VY protocol are provided in Table S1 and have been previously published [28]. The VY practice utilized a series of progressive videos adapted from the pre-recorded sequence shown during the initial experimental session. A “level 1” video was performed for week 1, a “level 2” video was performed for week 2, and a “level 3” video was performed for weeks 3 and 4 of the intervention. This was to ensure that all participants were provided with modifications and appropriate progressions of poses to make the VY practice accessible for a novice sample. At the initial supervised experimental session, a certified yoga instructor was present and encouraged the participants to continue making modifications as necessary for any of the poses. Participants were instructed to perform the 60-minute VY session three times per week for 4 weeks in the evening on non-consecutive days. Participants were instructed to maintain their usual daily activities throughout the intervention and to refrain from initiating any additional exercise programs.

Participants were provided with an HR monitor and watch (H10 and M200; Polar Electro Inc., Bethpage, New York) and instructed to wear the HR monitor during each yoga session to document adherence to the intervention. Each week, mean HR was calculated by averaging the HR across all yoga sessions completed during that week; each participant’s mean HR was presented as a percentage of their age-predicted maximum heart rate (APMHR; 208 – [0.7× age]) [30].

#### Non-active control condition

Participants randomized to the non-active control condition were asked to maintain their current lifestyle habits and refrain from starting a new exercise and/or yoga program. Participants were sent an online survey (Qualtrics; Provo, Utah) each week to document adherence. The survey asked whether there were any changes in their medical history or activity levels.

### Outcome measures

#### Sleep

##### Insomnia severity index

The ISI is a 7-item self-report questionnaire that assesses the severity of the nighttime and daytime impact of insomnia symptoms [23]. Scores range from 0 to 28, with higher scores indicating more severe insomnia symptoms [23, 31].

##### Actigraphy-assessed sleep

Data collected over eight nights from the Actiwatch were uploaded and edited in Actiware software (version 6.0.9). To limit potential bias, rest intervals were manually edited by a single trained staff member who followed standardized procedures that used a hierarchical method using multiple inputs: event markers, ambient light, sleep diary, and activity. Following editing of rest intervals, sleep/wake status for each epoch was determined with the Actiware algorithm using a wake threshold of 40 along with 5 minutes of sleep to establish sleep onset and 0 minutes to establish sleep offset [32]. Sleep efficiency (SE) and total sleep time (TST) were the primary actigraphy variables, with wake after sleep onset (WASO), sleep midpoint, bedtime, out-of-bed time, and total time in bed also processed and retained for secondary analysis.

##### Diary-assessed sleep

Data collected from the Expanded Consensus Sleep Diary were averaged across each time point for analysis. SE, TST, WASO, sleep midpoint, bedtime, out-of-bed time, time in bed, nocturnal awakenings, sleep quality, and feeling rested were processed and retained for secondary analysis.

#### Cardiovascular health

##### Blood pressure

The mean values of systolic BP and diastolic BP obtained during the daytime CVH assessment were used for data analysis.

##### Heart rate variability

Both daytime and nocturnal HRV data were downloaded from ActiLife6 software (ActiGraph Corp, Pensacola, Florida) as interbeat R–R intervals. For daytime HRV data, the first 5 minutes of valid data during the 6-minute recording during supine rest were used for analysis. For nocturnal HRV data, the interval used for analysis was aligned with the actigraphy-derived rest interval. Python (version 3.11.1) was used to pre-process the nocturnal data, creating sequential 5-minute segments throughout the entire rest interval. Any segment with a HR of 0 bpm (e.g. due to poor contact between the skin and electrodes of the HR sensor) was excluded from analysis. As done in previous work [33], HRV parameters were analyzed during each 5-minute segment throughout the night, with the mean across all 5-minute segments retained for analysis to standardize the recording duration and limit potential influence of sleep duration.

HRV data from each measurement source were imported as beat-to-beat RR intervals into Kubios HRV Premium software (version 3.5.0) for analysis [34]. Erroneous heartbeats indicated by abnormal RR interval values (including artifacts from ectopic beats and/or missed, extra, or misaligned beats) were detected and corrected by applying the automatic correction method. Both time- and frequency-domain variables were used in the statistical analyses. Root mean square of successive differences (RMSSD) was the primary measure of HRV in this study, as it is strongly linked to parasympathetic cardiac activation [35, 36]. Additional HRV measures were analyzed, including mean HR (bpm) and three frequency-domain measures: high frequency power (HF [0.15–0.40 Hz]), low frequency power (LF [0.04–0.15 Hz]), and LF/HF ratio.

##### Body composition

Using the resistance and reactance values from BIA, lean body mass was estimated using the equation by Segal et al. [37]. Body fat percentage was calculated as the difference between total body mass (measured previously at the baseline assessment) and lean body mass divided by the total body mass multiplied by 100 [37].

### Medical history, medication changes, and adverse events

A detailed medical history and current medications were obtained during baseline assessments. At the post-intervention assessment, participants were asked to report any changes in their medical history or medication use, including changes in medication dosage. Adverse events were passively collected by research staff based on voluntary reporting during any study-related contact with participants.

### Analytical approach

The estimated sample size was calculated using G^*^Power (version 3.1.9.6) with the ISI score as the primary outcome. Based on a previous study that examined the impact of yoga on sleep in a sample of adults with insomnia [38], this study aimed to detect an effect size improvement of *d* = 0.50 for the ISI. To achieve 80 per cent statistical power and an alpha level of 0.05, 26 participants (i.e. 13 participants per condition) were needed. To account for up to 20 per cent of attrition, we estimated that 32 participants would need to be randomized. All statistical analyses were performed using International Business Machines (IBM) Statistical Product and Service Solutions (SPSS) Statistics (v. 29.01.0; IBM Armonk, New York). Statistical significance was set at *p* < .05. Data are presented as mean ± standard error of the mean unless otherwise noted. Descriptive characteristics were compared between yoga and control groups. Independent samples *t*-tests were used for continuous variables, and chi-square tests were used for categorical variables. Normality was checked using the Shapiro–Wilk test for normality; in the case of violations of normality, the Wilcoxon Rank-Sum test was used as necessary.

All analyses were performed on an intention-to-treat basis, including all participants who were randomized in analyses using linear mixed models with an unstructured covariance matrix. The fixed effects within each model were group (yoga, control), time point (baseline, post-intervention), and group × time interaction, with participants included as random effects in each model. Linear mixed models can effectively account for missing data when missingness is assumed to be missing completely at random (i.e. unrelated to both observed and unobserved variables). To determine whether adherence was associated with treatment effects, per-protocol analyses were performed. This approach restricted analyses to only those participants who completed at least 10 of the 12 possible VY sessions and those in the control group who completed all four of the weekly check-in assessments.

Cohen’s *d* effect sizes were used to calculate the magnitude of difference for between-group comparisons and within-group comparisons following the intervention. Effect sizes were interpreted as small (*d* = 0.20), medium (*d* = 0.50), and large (*d* ≥0.80) [39].

Clinical significance was determined by comparing the yoga group to the control group for treatment response and remission rates at the end of the intervention. Treatment response was defined as an ISI score reduction from baseline to post-intervention of ≥8 points; remission was defined as an ISI score <8 at post-intervention [31]. Fisher’s exact tests were used to determine if rates of treatment response or remission differed between the yoga group and the control group.

## Results

### Participants

A total of 33 participants completed baseline assessments and were randomized into the study (yoga: *n* = 17; control: *n* = 16). Four participants withdrew from the study prior to completion, with 29 completing the study. No adverse events were reported during the trial. Figure 1 provides a flow diagram outlining participant retention throughout the trial.

**Figure 1:**
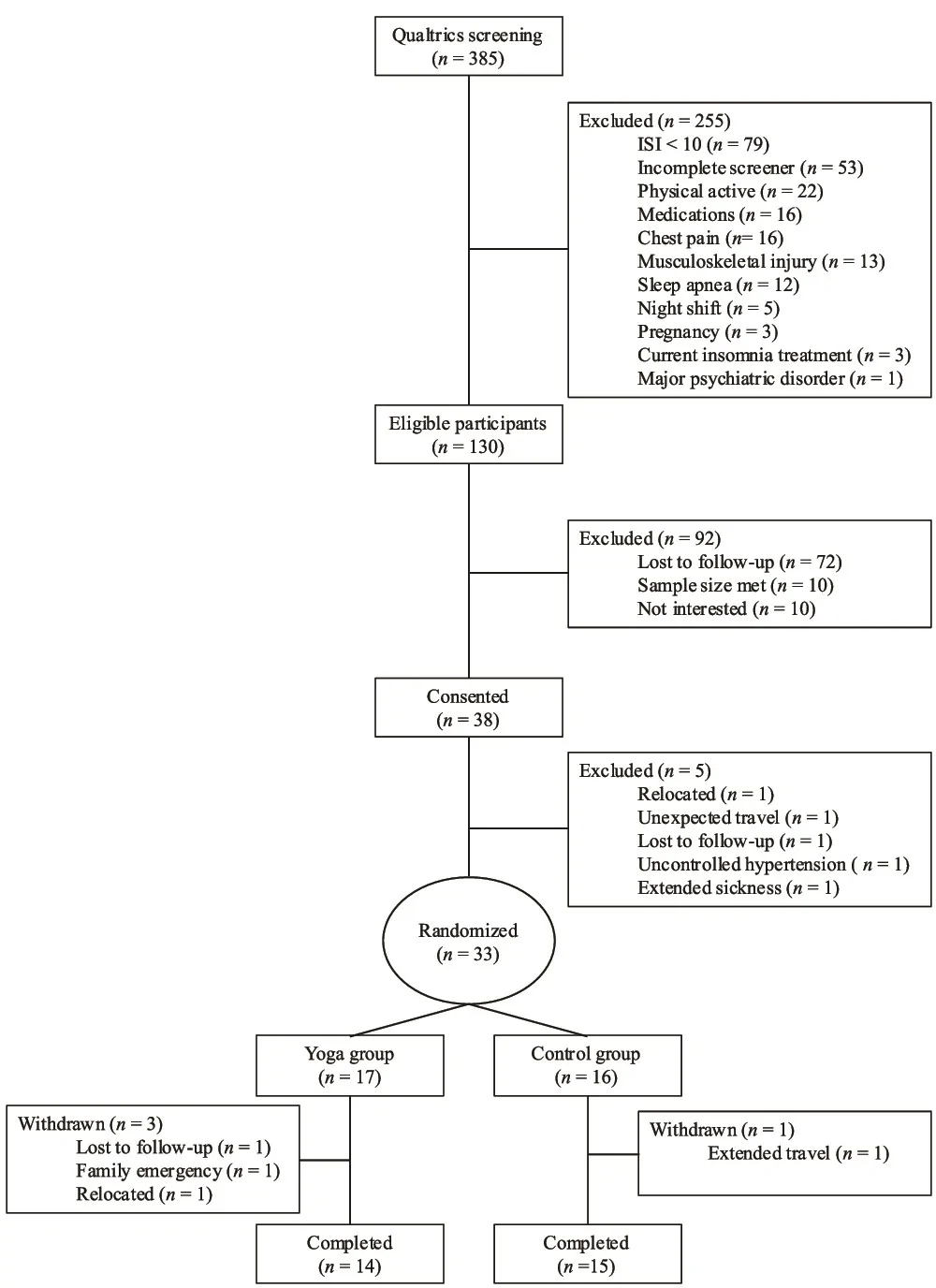
Summary of participant flow through the study.

Baseline participant characteristics are summarized in Table 1. The total sample (*N* = 33) had a mean ISI score of 15.88 ± 3.93, indicating moderate-severity insomnia symptoms on average. There were no significant differences between the two experimental groups at baseline (each *p* ≥ .14). One participant reported a change in medication dosage during the intervention period. Sensitivity analyses excluding the participant did not alter the study findings; therefore, results from the full sample are presented.

**Table 1:** Baseline participant characteristics

Characteristic	Full sample		Yoga group		Control group		Difference
Mean (SD)	*N* = 33		*n* = 17		*n* = 16		*P*-value
Age (years)	34.91	(10.64)	33.65	(10.91)	36.25	(10.52)	.491
Insomnia severity (ISI; 0-28)	15.88	(3.93)	15.18	(3.73)	16.63	(4.15)	.299
Apnea symptoms (STOP-Bang)	1.30	(0.92)	1.41	(1.13)	1.18	(0.79)	.709
Body mass index (kg/m^2^)	28.86	(7.19)	28.70	(8.58)	29.04	(5.64)	.606
*n* (%)							
Female sex	28	(84.84)	14	(82.35)	14	(87.50)	.680
Hispanic/Latino ethnicity	2	(6.06)	2	(11.76)	0	(0.00)	.144
Race							.796
White	26	(78.79)	14	(82.35)	12	(75.00)	
Black	4	(12.12)	2	(11.76)	2	(12.50)	
Asian	3	(9.09)	1	(5.88)	2	(12.50)	
Highest level of education							.366
Trade/technical	2	(6.06)	2	(11.76)	0	(0.00)	
Some college	6	(18.18)	3	(17.65)	3	(18.75)	
College graduate or higher	25	(75.76)	12	(70.59)	13	(81.25)	
Depression	13	(39.40)	7	(41.18)	6	(37.50)	.829
Generalized anxiety disorder	11	(33.33)	7	(41.18)	4	(25.00)	.325
Antidepressant/anxiolytic use	12	(36.4)	7	(41.18)	5	(31.31)	.721

All data are presented as mean (standard deviation) or *n* (%). Abbreviations: ISI = Insomnia Severity Index; SD = standard deviation.

### Intervention adherence

Adherence to the yoga intervention is presented in Table 2. On average, participants in the yoga group completed 9.38 ± 2.66 of the 12 prescribed yoga sessions; 7 of the 17 participants completed ≥10 of the 12 sessions. The mean HR during the VY session over the course of the 4 weeks was 102.51 ± 9.92 bpm, which was 55.25 per cent of the participants’ APMHR. The average practice time began at 17:41 hour. Participants in the control condition completed 3.75 ± 0.58 out of four potential check-ins; 13 of 16 participants completed all 4 weekly check-ins.

**Table 2:** VY intervention adherence characteristics

Characteristic	Week 1		Week 2		Week 3		Week 4		Total	
Mean (SD)										
Sessions per week	2.54	(0.78)	2.23	(1.01)	2.23	(0.93)	2.38	(0.86)	2.35	(0.81)
Session HR (bpm)	98.80	(9.80)	102.92	(12.33)	103.37	(10.01)	105.27	(10.07)	102.51	(9.92)
% APMHR (%)	53.34	(4.92)	55.72	(6.31)	56.09	(5.21)	56.85	(4.49)	55.25	(5.08)
Session duration (min)	56.97	(3.35)	56.42	(2.00)	56.98	(8.60)	57.95	(5.15)	57.15	(3.30)
Time of day (hh:mm)	17:14	(2:37)	17:40	(2:35)	18:06	(2:20)	17:48	(2:48)	17:41	(2:10)

%APMHR calculated using Tanaka formula [30]. Abbreviations: APMHR = age-predicted maximum heart rate; bpm = beats per minute; min = minutes; HR = heart rate; SD = standard deviation. Time of day is expressed in military time (hh:mm).

### Changes in insomnia severity

The change in ISI score from baseline to post-intervention did not significantly differ between the two groups (*F*(1, 29.57) = 2.71, *p* = .11). However, the ISI scores showed a significant main effect for both time (*p* < .001) and group (*p* < .03); the yoga group had significantly lower ISI scores across both time points, and ISI scores across both groups were significantly lower at post-intervention than at baseline. Within-group effect size estimates demonstrated a large reduction in ISI scores for the yoga group (*d* = –1.32) and a medium-sized reduction in ISI scores for the control group (*d* = –0.70); when compared between groups, a medium-sized reduction in ISI was observed for the yoga group relative to the control group (*d* = –0.63) (Figure 2A).

**Figure 2:**
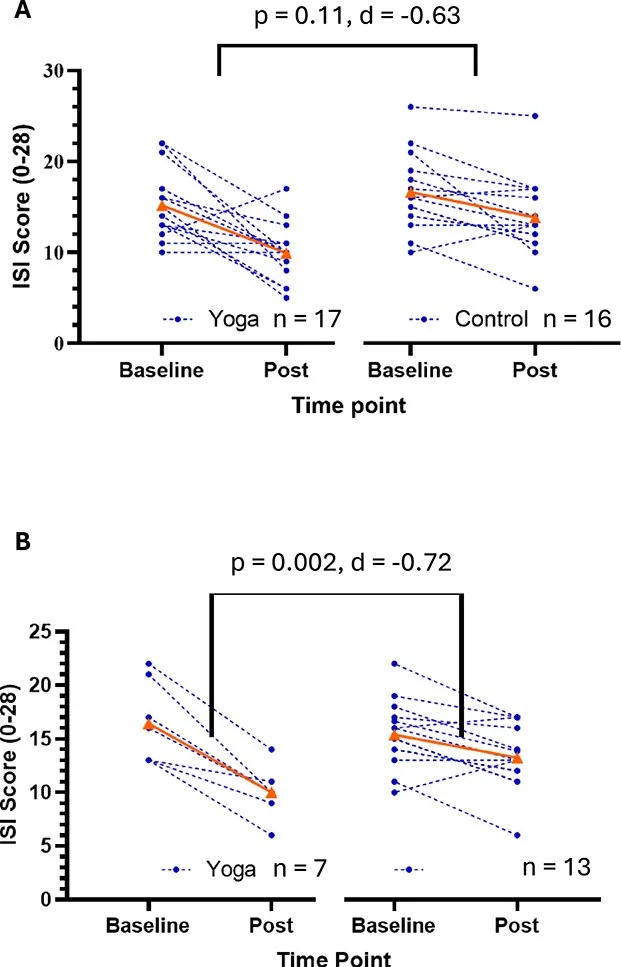
(A and B) Change in ISI scores from pre- to post-intervention. Significant differences are marked (*p* < .05). Cohen’s *d* effect sizes reflect between-group differences (yoga vs. control). Individual scores are shown in dotted lines; group means are indicated by the solid line for (A) intention-to-treat and (B) perprotocol analyses, defined as ≥10 of 12 yoga sessions completed or all 4 weekly check-ins for control.

When restricting the sample to per-protocol analyses based on those with high adherence to the protocol (*n* = 7 yoga, *n* = 13 control), the reduction in ISI from baseline to post-intervention was significantly greater for the yoga group compared to the control group (*F*(1, 18) = 12.53, *p* = .002) (Figure 2B).

In terms of treatment response, 5 of the 17 participants in the yoga group and 1 of the 16 participants in the control group met the criterion of an ≥8-point ISI reduction (*p* = .08). In terms of remission, 4 of the 17 participants in the yoga group and 1 of the 16 participants in the control group met the criterion of an ISI score <8 at post-intervention (*p* = .17).

### Changes in actigraphy- and diary-assessed sleep

None of the actigraphy- or diary-assessed sleep variables exhibited significant changes between groups over time (each *p* ≥ .06) (Tables 3 and 4).

**Table 3:** Pre- to post-intervention changes in actigraphy-assessed sleep variables

Variable	Baseline		Post		Time effect	Group effect	Interaction effect	Effect size
Mean (SEM)					*P*-value	*P*-value	*P*-value	Cohen’s *d*
SE (%)					.412	.996	.899	–0.20
Yoga	88.40	(1.43)	88.90	(1.06)				0.08
Control	88.30	(1.48)	89.99	(1.06)				0.28
TST (min)					.880	**.042**	.448	–0.23
Yoga	454.72	(15.84)	445.80	(17.44)				–0.14
Control	401.28	(16.33)	407.26	(17.31)				0.09
WASO (min)					.568	.348	.505	–0.15
Yoga	51.03	(6.45)	47.39	(4.68)				–0.14
Control	41.88	(6.65)	42.17	(4.70)				0.01
SOL (min)					.540	.972	.790	–0.07
Yoga	9.82	(2.71)	8.57	(1.77)				–0.11
Control	9.34	(2.80)	8.84	(1.76)				–0.04
Sleep midpoint (h)					.260	.582	.149	0.08
Yoga	3:58	(0:21)	3:41	(0:21)				–0.07
Control	4:05	(0:22)	4:07	(0:20)				0.00
Bedtime (h)					.193	.532	.613	–0.09
Yoga	23:36	(0:23)	23:25	(0:23)				–0.11
Control	24:14	(0:24)	24:13	(0:24)				–0.01
Out-of-bedtime (h)					.738	.261	.059	–0.31
Yoga	8:11	(0:22)	7:48	(0:22)				–0.25
Control	7:46	(0:63)	7:52	(0:22)				0.07
Time in bed (min)					.747	**.024**	.378	–0.26
Yoga	515.57	(16.98)	503.01	(17.95)				–0.18
Control	452.50	(17.51)	458.36	(17.81)				0.08

All data are presented in mean (SEM). Significant *p*-values are bolded (*p* < .05). Effect sizes are calculated as Cohen’s *d*, representing the magnitude of change from baseline to post-intervention. Effect sizes are shown for between-group (yoga compared to control) comparisons (top row) and within-group for each condition (bottom two rows). Abbreviations: SE = sleep efficiency; SEM = standard error of the mean; SOL = sleep onset latency; TST = total sleep time; WASO = wake after sleep onset. Time is expressed in military time (hh:mm).

**Table 4:** Pre- to post-intervention changes in diary-assessed sleep variables

Variable	Baseline		Post		Time effect	Group effect	Interaction effect	Effect size
Mean (SEM)					*P*-value	*P*-value	*P*-value	Cohen’s *d*
SE (%)					.254	.718	.281	0.30
Yoga	85.31	(4.84)	91.42	(3.54)				0.31
Control	86.34	(4.84)	86.51	(3.47)				0.01
TST (min)					**.007**	**.015**	.814	0.08
Yoga	432.87	(15.52)	466.71	(18.12)				0.51
Control	380.88	(15.52)	409.57	(17.72)				0.46
WASO (min)					.302	.424	.213	–0.53
Yoga	47.95	(11.05)	26.51	(5.32)				–0.47
Control	29.51	(11.06)	31.56	(5.85)				0.05
Awakenings					.713	.253	.238	–0.66
Yoga	2.01	(0.35)	1.40	(0.41)				–0.42
Control	1.99	(0.34)	2.32	(0.45)				0.24
SOL (min)					.766	.810	.249	–0.42
Yoga	32.07	(5.02)	26.64	(7.98)				–0.26
Control	29.82	(5.02)	33.05	(7.74)				0.16
Sleep midpoint (h)					.235	.846	.282	–0.18
Yoga	3:43	(0:22)	3:26	(0.:21)				–0.20
Control	3:41	(0:22)	3:40	(0:22)				–0.01
Bedtime (h)					.243	.076	.177	–0.11
Yoga	23:38	(0:21)	23:17	(0:21)				–0.25
Control	24:04	(0:22)	23:53	(0:22)				–0.13
Out-of-bedtime (h)					.789	**.036**	.895	–0.06
Yoga	7:56	(0:29)	7:52	(0:22)				–0.04
Control	7:08	(0:28)	6:57	(0:24)				–0.10
Time in bed (min)					.243	.076	.177	–0.80
Yoga	515.08	(19.05)	513.12	(19.12)				–0.02
Control	454.55	(19.50)	480.76	(18.81)				–0.83
Sleep quality (1–5)					.586	.072	.121	0.79
Yoga	2.83	(0.23)	3.33	(0.21)				0.53
Control	2.81	(0.23)	2.57	(0.22)				–0.26
Feeling rested (1–5)					.852	**.002**	.276	0.53
Yoga	2.65	(0.22)	2.93	(0.22)				0.31
Control	2.13	(0.23)	1.93	(0.236)				–0.22

All data are presented in mean (SEM). Significant values are bolded (*p* < .05 Effect sizes are calculated as Cohen’s *d*, representing the magnitude of change from baseline to post-intervention. Effect sizes are shown for between-group (yoga compared to control) comparisons (top row) and within-group comparisons for each condition (bottom two rows). Abbreviations: SE = sleep efficiency; SEM = standard error of the mean; SOL = sleep onset latency TST = total sleep time; WASO = wake after sleep onset. Time is expressed in military time (hh:mm).

### Changes in nocturnal HRV

The change in nocturnal RMSSD HRV from baseline to post-intervention did not significantly differ between the two groups (*F*(1, 25.06) = 0.47, *p* = .50). When restricting the sample to per-protocol analyses, results did not differ (i.e. group × time interaction, *p* > .47). For each of the additional nocturnal HRV parameters that were examined (HR, LF HRV, HF HRV, and LF/HF ratio), changes in these variables from baseline to post-intervention were similar between the two groups (i.e. each group × time *p* ≥ .37) (Table 5).

**Table 5:** Pre- to post-intervention changes in nocturnal and daytime CVH

Variable	Baseline		Post		Time effect	Group effect	Interaction effect	Effect size
Mean (SEM)					*P*-value	*P*-value	*P*-value	Cohen’s *d*
Nocturnal CVH outcomes								
HR (bpm)					.584	.457	.994	–0.00
Yoga	70.34	(2.28)	70.98	(2.43)				0.07
Control	67.97	(2.35)	68.59	(2.44)				0.07
RMSSD (ms)					.664	.728	.501	0.11
Yoga	39.46	(6.79)	40.02	(7.39)				0.02
Control	37.57	(7.00)	34.96	(7.52)				–0.09
LF (log)					.510	.846	.653	0.12
Yoga	6.31	(0.23)	6.31	(0.24)				0.00
Control	6.20	(0.24)	6.06	(0.24)				–0.10
HF (log)					.339	.580	.373	0.07
Yoga	5.72	(0.35)	5.70	(0.35)				–0.01
Control	5.68	(0.36)	5.56	(0.56)				–0.08
LF/HF ratio					.589	.386	.536	–0.10
Yoga	2.82	(0.38)	2.97	(0.43)				0.06
Control	2.41	(0.40)	2.40	(0.43)				–0.01
Daytime resting CVH outcomes								
Systolic BP (mmHg)					.158	.684	.302	0.22
Yoga	115.06	(2.92)	114.56	(2.47)				–0.04
Control	114.88	(3.00)	111.74	(2.49)				–0.26
Diastolic BP (mmHg)					.195	.997	.298	0.21
Yoga	67.94	(2.52)	67.67	(1.98)				–0.03
Control	69.00	(2.60)	66.59	(2.00)				–0.23
HR (bpm)					.866	.827	**.047**	–0.53
Yoga	72.41	(2.47)	69.50	(2.60)				–0.29
Control	70.47	(2.54)	72.93	(2.60)				0.24
RMSSD (ms)					.459	.684	.755	0.13
Yoga	52.19	(14.85)	47.23	(8.67)				–0.08
Control	50.99	(13.38)	38.83	(7.61)				–0.24
LF (log)					.874	.718	.646	–0.19
Yoga	6.67	(0.39)	6.47	(0.35)				–0.12
Control	6.38	(0.38)	6.47	(0.31)				0.06
HF (log)					.990	.634	.843	0.09
Yoga	6.30	(0.41)	6.24	(0.47)				–0.04
Control	6.00	(0.37)	6.08	(0.42)				0.05
LF/HF ratio					.811	.336	.579	–0.27
Yoga	1.81	(0.40)	1.50	(0.52)				–0.19
Control	2.08	(0.36)	2.20	(0.47)				0.08
Body fat percent					.416	.465	.449	0.11
Yoga	35.25	(2.31)	35.21	(2.46)				0.00
Control	38.25	(2.38)	37.14	(2.51)				–0.12

All data are presented in mean (SEM). Significant *p*-values are bolded (*p* < .05). Effect sizes are calculated as Cohen’s *d*, representing the magnitude of change from baseline to post-intervention. Effects are shown for between-group comparisons (yoga compared to control) (top row) and within-group comparisons for each condition (bottom two rows).

Abbreviations: bpm = beats per minute; HF = high frequency power; HR = heart rate; LF = low frequency power; LF/HF ratio = low frequency to high frequency ratio; ms = millisecond; RMSSD = root mean square successive difference; SEM = standard error of the mean.

### Changes in daytime CVH

Pre- and post-intervention daytime resting CVH values are presented in Table 5. Of these variables, only resting HR demonstrated a significant group × time interaction (*F*(1, 27.97) = 4.30, *p* = .047), indicating that the reduction in resting HR from baseline to post-intervention was significantly greater in the yoga group compared to the control group. The reduction in resting HR between groups was medium-sized in magnitude (*d* = –0.53).

## Discussion

The present study examined the effects of a 4-week VY intervention on sleep- and cardiovascular-related outcomes in 33 adults with self-reported insomnia symptoms to address the knowledge gap regarding the short-term effects of VY on sleep and CVH. Our findings partially support our main hypothesis that 4 weeks of VY would significantly reduce insomnia symptoms relative to a control condition.

Our primary intent-to-treat analyses failed to observe a significant difference in ISI change between the yoga and control groups. However, per-protocol analyses suggested a greater reduction in ISI scores for highly adherent yoga participants than for highly adherent control participants. In addition, despite the non-significant intent-to-treat findings, we observed large (*d* = –1.23) and medium (*d* = –0.63) effect sizes for within- and between-group comparisons following the intervention, indicating that the yoga group had a large magnitude of reduction in ISI scores at follow-up that was moderately larger than the control group. These results align with previous findings from Khalsa and Goldstein [38] and Afonso et al. [40], who observed medium to large effect size reductions for ISI following a yoga intervention in adults with insomnia disorder. Similarly, Taibi and Vitiello [41] found significant reductions in ISI scores following an 8-week gentle evening yoga practice in women with osteoarthrosis who reported sleep complaints. Only one other study examined a higher intensity form of yoga that was similar to VY. Papp et al. observed a significant association between ISI change and the total yoga dose (*r* = 0.55) among those who practiced between 5 and 10 hours over the 6-week intervention [42]. While only a 4-week intervention, our study prescribed a total of 12 hours of yoga over 4 weeks; in per-protocol analyses of the most adherent participants, we found that the yoga intervention led to a significantly greater ISI reduction than the control group. Our study highlights the importance of regular engagement in VY, as we observed that participants who were highly adherent to the yoga intervention exhibited statistically lower ISI scores.

Our results are promising because the yoga group experienced large within-group reductions in insomnia severity after a brief 4-week intervention. The observed reduction in ISI scores in the yoga group is clinically significant. Approximately 30 per cent of the yoga participants were categorized as treatment responders (i.e. ≥8-point ISI reduction) compared with 6 per cent of the control group. A similar pattern was observed for remission rates: 23.5 per cent of the yoga participants achieved remission (i.e. post-intervention ISI <8) compared with 6.3 per cent of participants in the control group. These treatment response and remission rates are lower than those documented by Khalsa and Goldstein, who showed that 52.6 per cent of yoga participants had at least an 8-point reduction in ISI total scores and 47.4 per cent of yoga participants achieved remission. However, Khalsa and Goldstein recruited only those with a diagnosis of insomnia, their baseline ISI scores were significantly higher than those in our study (i.e. 17.8 vs. 15.9), and their intervention lasted 8 weeks in comparison with our 4-week intervention [38]. Therefore, a longer intervention in a sample with greater insomnia severity may lead to a higher percentage of yoga participants demonstrating a treatment response and/or remission.

Our secondary sleep-related outcomes examined the effects of the VY intervention compared with a control group on actigraphy-assessed SE and TST. Neither SE nor TST changed following the intervention relative to the control group. Baseline mean actigraphy-assessed SE and TST for the sample were 88.4 per cent and 7.1 hours, respectively, exceeding the thresholds of ≥85 per cent and ≥7 hours that are often recommended for optimal sleep health [43]; therefore, leaving limited room for further improvement.

Understanding that actigraphy-assessed sleep often does not reflect subjective sleep in insomnia patients [44], we also examined pre- to post-intervention changes in diary-assessed sleep. We observed medium within-group effect sizes for several variables generally associated with sleep consolidation and quality, indicating clinically meaningful increases in SE, TST, and overall sleep quality, while medium effect sizes also indicated clinically meaningful reductions in WASO, the frequency of awakenings, and sleep onset latency (SOL) in the yoga group over time. These results are of particular importance because insomnia is commonly characterized as a subjective sleep disorder marked by chronic dissatisfaction with an individual’s sleep quality or quantity [45]. This supports findings of prior research involving samples with sleep disturbances who assessed sleep using diaries. Our study found an approximate 6 per cent increase in diary-assessed SE, while Khalsa and Goldstein reported an approximate 10 per cent increase in SE. This discrepancy may be attributed to the fact that the participants in our study began with a slightly higher diary-assessed SE at baseline (85.8 per cent) than Khalsa and Goldstein (77.2 per cent), allowing for less potential for improvement in our sample [38]. In addition, the shorter duration of intervention in our study (4 versus 8 weeks) may have limited the magnitude of improvement in these outcomes.

The secondary aim of this study was to examine the effects of VY on nocturnal and daytime CVH outcomes. To our knowledge, this is the first study to examine the effects of yoga on nocturnal HRV. However, our nocturnal and daytime HRV results do not align with previous studies showing that yoga can improve both time- and frequency-domain HRV parameters that are assessed during the daytime [17, 18]. Exercise training is well known to improve HRV parameters; however, research involving aerobic exercise has previously suggested that increased vagal activity may not be observed until approximately 3 months of regular training has been achieved [46]. Instead, our findings align more closely with those of Posadzki et al. In their meta-analysis, they failed to observe convincing evidence that yoga improves HRV in healthy adults or those with chronic conditions [47].

The only CVH outcome that significantly improved relative to the control condition over time was daytime resting HR, with a ~3-bpm reduction for the yoga group alongside a ~2.5-bpm increase in the control condition. Our results are similar to a study conducted by Kaleeswari et al. in their 6-week yoga intervention administered to women with no prior yoga experience, as they observed an approximately 3-bpm reduction in resting HR at post-intervention [48]. Our results also align with previously published meta-analyses by Cramer et al. and Chu et al., who found a 7- and 5-bpm decrease in resting HR, respectively, following yoga practice [49, 50]. Nonetheless, according to Cramer et al., the most prominent effects in improving cardiovascular risk factors were observed after approximately 12 weeks of regular yoga training [51]. Additionally, it was previously suggested by Cramer et al. that yoga breathing interventions may be more effective than yoga with physical postures, although the studies included in this meta-analysis were deemed low-quality evidence [52]. To our knowledge, only one other study has examined the effects of a VY intervention on CVH. In a pilot study of 23 adults with type 2 diabetes, Yang et al. demonstrated medium- to large-sized improvements for weight, BP, lipids, insulin, and fasting glucose levels following 3 months of VY when compared to an education group [53]. Our results suggest that short-term VY training (≤4 weeks) may not be a sufficient dose to elicit widespread significant improvements in CVH.

Yoga may improve sleep through multiple pathways. VY may improve sleep through its status as a form of physical activity. Exercise is a common behavioral recommendation to improve sleep [54]. Consistent engagement in exercise can significantly reduce SOL and WASO, and increase TST. Regular exercise has also been demonstrated to improve cardiac and autonomic control by decreasing resting HR and enhancing parasympathetic nervous system activity [55]. Additionally, Chu et al. reported that yoga elicited comparable effects on multiple cardiometabolic outcomes (e.g. BP, HR, body mass index, lipids) relative to aerobic exercise, suggesting the possibility of similar underlying mechanisms [50]. Another potential pathway through which yoga may improve sleep is through reductions in symptoms of stress, anxiety, and/or depression, each of which is an independent risk factors and/or symptoms of insomnia [56]. Multiple studies have shown that both a single session of yoga and regular yoga training can reduce self-reported and physiological markers of stress [18, 57, 58]. Furthermore, a 12-week yoga intervention led to greater improvements in mood and anxiety symptoms than a metabolically matched walking exercise intervention [59]. When performed consistently over time, the meditative component of yoga contributes to enhanced self-regulation, emotional stability, and resilience to stress [8]. Therefore, enhancements in mood, reductions in anxiety, depression, and stress levels may create additional avenues through which yoga can positively impact sleep.

### Strengths and limitations

This study has many strengths that address the limitations of the current literature. First, our study implemented a VY protocol that has been previously validated to meet the criteria associated with moderate-intensity physical activity [14, 15]. The protocol was standardized using pre-recorded videos that included demonstration and verbal cues, and the yoga sessions were completed in participants’ homes, increasing the generalizability of the intervention. Adherence was also objectively documented. We recruited participants with complaints of insomnia symptoms and assessed sleep using actigraphy, sleep diaries, and validated questionnaires. Finally, the randomized controlled trial design enabled examination of both within- and between-changes due to the experimental interventions.

Despite these strengths, it is important to recognize the study’s limitations. One of the main limitations were the small sample size and the predominance of White and female participants, which may restrict the generalizability of our findings. In a related fashion, this study conducted multiple analyses without any statistical adjustment for multiple comparisons, thereby increasing the potential for Type I error. Although we recruited participants with insomnia symptoms, they were not formally diagnosed with insomnia disorder; this may have contributed to the lack of significant impairment in sleep at baseline and, as a result, limited room for improvement. Additionally, participants with depression, anxiety, or cardiovascular risk factors were not excluded, and these factors may independently influence sleep and cardiovascular outcomes. Although depression, anxiety, and medication use did not differ between groups at baseline, the small sample size limits our ability to examine the potential influence on observed outcomes. Another limitation of our study pertained to monitoring adherence to the yoga intervention. While the use of HR monitors and watches provided timestamps and HR recordings for each home-based yoga practice, we relied on participants wearing the watch during each session. Additionally, adherence data were reviewed only at the conclusion of the intervention; this prevented us from encouraging greater engagement with those who were initially non-adherent. Lastly, the present study was not designed to determine whether changes in insomnia symptoms were mediated by physiological adaptations to physical activity, psychological factors, or interactions between these pathways.

Given the established efficacy of CBT-I, the present findings may represent a complementary behavioral approach for improving sleep and CVH in individuals experiencing insomnia symptoms. However, future investigations should examine the impact of a more prominent dose of VY on sleep in a larger sample with diagnosed insomnia. Additionally, future studies should explore whether sleep changes more gradually over time and whether sleep changes on a night-to-night basis due to yoga practice. This recommendation is supported by participants’ open-ended post-intervention comments indicating that they slept better on nights in which they practiced yoga in an open-ended comment from post-intervention.

## Conclusion

Overall, despite non-significant improvements in any sleep parameters from the primary linear mixed model analyses, our pilot study observed promising improvements in self-reported sleep outcomes (i.e. ISI, diary-assessed sleep) following a 4-week VY intervention compared to control in a sample of adults with insomnia symptoms. Nevertheless, these results suggest that a short-term intervention of VY has minimal impact on CVH but may reduce insomnia severity among those who are highly adherent.

## Supplementary Material

Manuscript_Tables_zpag107

## Data Availability

The data and study materials are available from the corresponding author upon reasonable request.
