# Supplementary material for: Effects of a 4-week vinyasa yoga intervention on sleep and cardiovascular health in adults with insomnia symptoms: a pilot randomized controlled trial
Source: Sleep Adv. 2026 Oct 7;7(4):zpag107. doi: 10.1093/sleepadvances/zpag107 (PMC13744281; doi:10.1093/sleepadvances/zpag107)
Supplement: Supplementary file 1 [file manuscript_tables_zpag107.docx]

Supplementary Table 1. Vinyasa Yoga Protocol

| **Time/ Sequence (minutes)** | **Asana 1** | **Asana 2** | **Asana 3** | |
| --- | --- | --- | --- | --- |
| **Sequence** | **Integration** | | | |
| 0:00-1:00 | Child's pose** | Down Dog** | | |
| 1:01-2:00 | 3-Legged Dog Right | 3-Legged Dog Left | | |
| 2:01-3:00 | Ragdoll | Mountain | Hands to Heart Center | |
| **Sequence** | **Sun Salutations** | | | |
| 3:01-4:00 | Sun A *** | | | |
| 4:01-5:00 | Sun A 2*** | | | |
| 5:01-6:00 | Sun A 3*** | Sun A 4*** | | |
| 6:01-7:00 | Sun A 5*** | | | |
| 7:01-8:00 | Sun B 1 | | | |
| 8:01-9:00 | Sun B 1*** | | | |
| 9:01-10:00 | Sun B 2*** | | | |
| 10:01-11:00 | Sun B 3*** | | | |
| 11:01-12:00 | Sun B 3*** | | | |
| 12:01-13:00 | Sun B 4*** | | | |
| 13:01-14:00 | Sun B 5*** | | | |
| **Sequence** | **Crescent Lunge Series** | | | |
| 14:01-15:00 | Flip Dog Right* | Side Plank Right* | | |
| 15:01-16:00 | Crescent Lunge Right | Crescent Lunge Twist Right | | |
| 16:01-17:00 | Warrior II | Extended Side Angle* | | |
| 17:01-18:00 | Flip Dog Left | Side Plank Left | | |
| 18:01-19:00 | Crescent Lunge Left | Crescent Lunge Twist Left | | |
| 19:01-20:00 | Warrior II | Extended Side Angle* | | |
| 20:01-21:00 | Chair | Chair Twist | | |
| 21:01-22:00 | Forward Fold** | Crow* | | |
| 22:01-23:00 | Chair | | | |
| 23:01-24:00 | Chair Twist | Forward Fold** | | |
| 24:01-25:00 | Crow* | | | |
| **Sequence** | **Balancing** | | | |
| 25:01-26:00 | Eagle Right | Eagle Left | Eagle Right | |
| 26:01-27:00 | Eagle Left | Leg Raise Right | | |
| 27:01-28:00 | Airplane Right | Half Moon Right | | |
| 28:01-29:00 | Leg Raise Left | Airplane Left | | |
| 29:01-30:00 | Half Moon Left | Dancer Right |  | |
| 30:01-31:00 | Dancer Left | Dancer Right | Dancer Left | |
| **Sequence** | **Standing Series** | | | |
| 31:01-32:00 | Tree Right | Tree Left* | | |
| 32:01-33:00 | Warrior II | Triangle Right | | |
| 33:01-34:00 | Wide Leg Fold | Pyramid Right | | |
| 34:01-35:00 | Twist Triangle Right | Warrior II | | |
| 35:01-36:00 | Triangle Left | Wide Leg Fold | | |
| 36:01-37:00 | Pyramid Left | Twist Triangle Left* | | |
| **Sequence** | **Back Bending Series** | | | |
| 37:01-38:00 | Locust 1 | Locust 2 | | |
| 38:01-39:00 | Bow 1 | Bow 2 | | |
| 39:01-40:00 | Camel 1 | Camel 2 | | |
| 40:01-41:00 | Bridge | Wheel 1 | | |
| 41:01-42:00 | Wheel 2 | Wheel 3 | | |
| 42:01-43:00 | Wheel 4 | Wheel 5 | | |
| 43:01-44:00 | Wheel 6 | Supine Butterfly | | |
| 44:01-45:00 | Happy Baby | Boat* | | |
| **Sequence** | **Restorative Series** | | | |
| 45:01-46:00 | Half Pigeon Right** | Double Pigeon Right** | | |
| 46:01-47:00 | Half Pigeon Left** | | | |
| 47:01-48:00 | Double Pigeon Left** | | | |
| 48:01-49:00 | Frog** | | | |
| 49:01-50:00 | Single Leg Right** | | | |
| 50:01-51:00 | Single Leg Left** | | | |
| 51:01-52:00 | Double Leg Extension** | Table Top** | | |
| 52:01-53:00 | Fish** | | | |
| 53:01-54:00 | Shoulder Stand** | | | |
| 54:01-55:00 | Plow** | Deaf Man’s Pose** | | |
| 55:01-56:00 | Supine Twist Right** | | | |
| 56:01-57:00 | Supine Twist Left** | | | |
| 57:01-58:00 | Seated Butterfly* | | | |
| 58:01-59:00 | Savasana | | | |
| 59:01-60:00 | Roll to Seat Right Side | Move to Seated position | | Hands to Heart Center |

Based on the *Journey into Power* sequence from Baptiste Power Yoga; 5 breaths per pose were performed unless noted otherwise (5 breaths = 20 seconds).

Transitions, vinyasa (plank, low plan, cobra/upward-facing dog) to downdog.

**10 breaths

***1 breath
